# P2Rank: machine learning based tool for rapid and accurate prediction of ligand binding sites from protein structure

**DOI:** 10.1186/s13321-018-0285-8

**Published:** 2018-08-14

**Authors:** Radoslav Krivák, David Hoksza

**Affiliations:** Department of Software Engineering, Charles University, Prague, Czech Republic

**Keywords:** Ligand binding sites, Protein pockets, Binding site prediction, Protein surface descriptors, Machine learning, Random forests

## Abstract

**Background:**

Ligand binding site prediction from protein structure has many applications related to elucidation of protein function and structure based drug discovery. It often represents only one step of many in complex computational drug design efforts. Although many methods have been published to date, only few of them are suitable for use in automated pipelines or for processing large datasets. These use cases require stability and speed, which disqualifies many of the recently introduced tools that are either template based or available only as web servers.

**Results:**

We present P2Rank, a stand-alone template-free tool for prediction of ligand binding sites based on machine learning. It is based on prediction of ligandability of local chemical neighbourhoods that are centered on points placed on the solvent accessible surface of a protein. We show that P2Rank outperforms several existing tools, which include two widely used stand-alone tools (Fpocket, SiteHound), a comprehensive consensus based tool (MetaPocket 2.0), and a recent deep learning based method (DeepSite). P2Rank belongs to the fastest available tools (requires under 1 s for prediction on one protein), with additional advantage of multi-threaded implementation.

**Conclusions:**

P2Rank is a new open source software package for ligand binding site prediction from protein structure. It is available as a user-friendly stand-alone command line program and a Java library. P2Rank has a lightweight installation and does not depend on other bioinformatics tools or large structural or sequence databases. Thanks to its speed and ability to make fully automated predictions, it is particularly well suited for processing large datasets or as a component of scalable structural bioinformatics pipelines.

**Electronic supplementary material:**

The online version of this article (10.1186/s13321-018-0285-8) contains supplementary material, which is available to authorized users.

## Background

### Motivation

Prediction of ligand binding sites (LBS, or simply pockets^1^) from protein structure has many applications in elucidation of protein function [1] and rational drug design [2–4]. It has been employed in drug side-effects prediction [5], fragment-based drug discovery [6], docking prioritization [7, 8], structure based virtual screening [9] and structure-based target prediction (or so called inverse virtual screening) [10]. Increasingly, LBS prediction is being used in large-scale structural studies that try to analyze and compare all known and putative binding sites on a genome-wide level [11–15]. In practice, it is often the case that predicting ligand binding sites is not an end in itself but it represents only a step in larger automated solution or pipeline. For example, druggability prediction server PockDrug-Server [16] relies on LBS prediction internally. Similarly, allosteric site prediction tools Allosite [17] and AlloPred [17] both employ pocket prediction tool Fpocket [18] as the first step of their algorithms.

In the rest of this section we will summarize existing methods and available tools. We will introduce categorization along several lines:

1. web servers/stand-alone tools,
2. template based/template-free methods,
3. residue-centric/pocket-centric prediction,

and we will discuss strengths and weaknesses of tools in these categories. We will also discuss an overlooked aspect of the speed of available tools. We will try to convey that there is a strong case for new fast stand-alone user-friendly tool that is not based on search in a large template library of known protein-ligand complexes.

### Existing approaches

Existing methods for LBS prediction are based on variety of algorithmic approaches. Traditionally, methods have been categorized based on their main algorithmic strategy into geometric, energetic, conservation based, template based (the last two also sometimes referred to as evolutionary) and machine learning/knowledge based. In reality, many of the state-of-the-art tools are based on some combination of the mentioned approaches. Methods based on consensus of results of other algorithms have also emerged. Table 1 lists available tools for LBS prediction from protein structure introduced since 2009 (to cover most recent and still widely used methods). In the following paragraphs we will introduce the tools that we have used to comparatively evaluate the performance of P2Rank. More details on existing approaches, including older ones, can be found in numerous reviews and surveys [3, 7, 19–25].

Fpocket is a fast geometric stand-alone tool based on filtering and clustering of alpha spheres found by way of Voronoi tessellation [18]. It has been one of the most widely used methods in recent years, especially in large scale applications. Fpocket typically produces relatively high number of predicted pockets for one protein. Among them, Fpocket finds most of the known binding sites, but they are not always ranked at the top. To address this problem, we have previously developed a method called PRANK [26] that is able to re-score binding site predicted by Fpocket and thus improve relevance of its results (i.e. improve identification success rate among Top-n pockets). Usage simplicity of Fpocket together with its computational efficiency contribute to the fact that it remains a popular choice for LBS prediction, as can be illustrated by its employment in recent large-scale structural studies [11–15]. Overall good user experience with Fpocket in contrast with other available methods has been an inspiration for designing our tool.

MetaPocket 2.0 is a prominent example of a consensus based method [27]. It aggregates results produced by 8 different previously published algorithms by taking top 3 sites predicted by each method. It was shown to perform better that any single one of those individual methods. MetaPocket 2.0 is only available as a web server.

SiteHound is one of the latest energetic methods, and the latest one with stand-alone version [28]. It works by placing a probe on a grid points around a protein surface and calculating interaction energies with the help of underlying force field software. It is available as a web server and as a fully automated stand-alone tool.

Fpocket, SiteHound and MetaPocket 2.0 belong to the most cited and widely used template-free methods introduced in the last decade.

The tool presented in this article is based on machine learning from examples. As a main approach, machine learning has been under-utilized among published methods. Although some studies that applied machine learning to the problem have been published, their focus was mainly on classification of binding residues rather than on predicting binding sites as such [29–31]. Machine learning has been also employed to solve partial tasks in complex eFindSite and COACH methods. Tools based primarily on machine learning have been introduced only very recently [32, 33] (with notable earlier exception [34]). The latest one of them is DeepSite, a method based on multi-layer (for different atom types) voxelized representation of 3D space and deep convolutional neural networks. It is available only as a web server, but it is reasonably fast and has usable, although undocumented web API.

Studies that introduced existing methods reported relatively high identification success rates, usually on traditional small datasets. However, the results of the only independent benchmark [21] suggest that existing methods may not be as accurate as previously believed when applied to new datasets. It showed that there is still a need for more accurate methods, and that nominally high results reported by the authors of respective methods may not be always indicative of their true performance on unseen proteins.

**Table 1:** Availability of existing tools for ligand binding site prediction from protein structure introduced since 2009

Name	Year	Type	Web server	Stand-alone	Fully automated^†^	Source Code
SiteMap [35]	2009	Geometric	–	Yes	Yes	–
Fpocket [18]	2009	Geometric	Yes	Yes	Yes	Yes
SiteHound [28]	2009	Energetic	Yes	Yes	Yes	Yes
ConCavity [36]	2009	Conservation	Yes	Yes	–	Yes
3DLigandSite [37]	2010	Template	Yes	–	–	–
POCASA [38]	2010	Geometric	Yes	–	–	–
DoGSite [39]	2010	Geometric	Yes	–	–	–
MetaPocket 2.0 [27]	2011	consensus	Yes	–	–	–
MSPocket [81]	2011	Geometric	–	Yes	Yes	Yes
FTSite [40]	2012	Energetic	Yes	–	–	–
LISE [41]	2012	Knowledge/conservation	Yes	Yes	–	–
COFACTOR [42]	2012	Template	Yes	Yes	Yes	–
COACH [43]	2013	Template^†^^†^	Yes	Yes	Yes	–
G-LoSA [44]	2013	Template	–	Yes	–	Yes
eFindSite [45]	2013	Template	Yes	Yes	–	Yes
GalaxySite [46]	2014	Template/docking	Yes	–	–	–
LIBRA [47]	2015	Template	Yes	Yes	–	–
P2Rank (this work)	2015*	Machine learning	–**	Yes	Yes	Yes
bSiteFinder [48]	2016	Template	Yes	–	–	–
ISMBLab-LIG [32]	2016	Machine learning	Yes	–	–	–
DeepSite [33]	2017	Machine learning	Yes	–	–	–

^†^Applies to stand-alone versions

^††^Consensus of template based methods: TM-SITE, S-SITE and COFACTOR (also FINDSITE and ConCavity in web version)

*Algorithm introduced in conference proceedings [49]

**In development

### Stand-alone tools versus web servers

Relatively many methods for LBS prediction have been published to date, and it may seem that the field is crowded with tools available for researchers. However, after closer survey (see Table 1) we found that only few of the published methods are available as a stand-alone software that can be used locally (in contrast to web-only methods), and most of those that are are unnecessarily complicated to use (i.e. users are required to perform preprocessing tasks that could have been automated by the authors of the software). Even fewer of them are available as open source software.

The recent trend has been to make methods available only as a web server. Contrary to that, we believe that there is still a strong case for stand-alone tools. Online methods with a web interface have many advantages including usage simplicity, visual presentation and the fact that they are ready to be used without installation. They are best suited for use cases when researchers want to manually examine one or a small number of proteins. However, for many other use cases, such as those that involve processing of large datasets, tools need to be used in automated mode. Web-only tools are intended for interactive use and unfortunately, as a rule, do not provide stable and documented APIs. Thus, the only way how to use those tools in automated mode is to write patchy web scraping scripts that upload proteins and parse the result pages, which format is not well defined and can change without notice. This approach is far from ideal since it leads to fragile implementations and potentially irreproducible results. Another consideration when using web-only tools is a lack of control over employed computational resources and consequently over speed, stability and availability. Locally executable tools are therefore more suitable in many use cases such as batch processing of large datasets, or in cases where LBS prediction is needed as a stable part of a larger software solution or pipeline.

We believe that from the user perspective, predicting LBS with a stand-alone tool should be as simple as running a single command. With notable exception of Fpocket (fpocket -f protein.pdb), SiteHound and COACH, this is rarely the case. All other methods we examined were not able to produce predictions in fully automated manner, and required a manual multi-step procedure for either generating secondary data or data preprocessing of some sort. For example, methods based on sequence conservation like ConCavity or LigsiteCSC [50] ask user to calculate or download sequence conservation scores for a given protein first. Similarly, some template based methods like eFindSite (and also LISE) require pre-calculated sequence alignments as an input (in addition to other preprocessing steps).

Such requirements pose additional work to users and sometimes put them in front of decisions that they may not be ready to make (e.g. what is the best way to calculate conservation scores or which algorithm/database should be used to generate alignments). Tools that are not fully automated thus pose unnecessary usability barriers that can hinder their widespread adoption.

### Template based versus template-free methods

A substantial effort in the recent decade has been devoted to the development of template based methods, which exploit the general tendency of certain protein families to bind ligands at similar locations [45]. From earlier methods like ProFunc [51] and FINDSITE [52, 53] to the recent, more complex methods, their defining feature is that they all rely on a large databases of know protein-ligand complexes. This template database typically consists of a substantial portion of all protein-ligand complexes in the PDB. The difference between methods is in the sophistication by which they search in their template library and then align and aggregate results to form predictions. This search is usually done in a sequential manner, which accounts for the fact that they are typically much slower than template-free methods.

Template based methods belong to the most successful and practically useful of currently available methods. This is because for any unannotated protein, regardless of the use case, we would probably like to know the answer to the question: Are there any known examples of confirmed binding sites on related proteins? Template based methods can give (to some extent) definitive answer to this question, which can be very informative either way. They are able to produce high confidence predictions (especially when closely related proteins are found) supported by examples from the template library.

However, apart from slow speed, template based methods have a fundamental theoretical limitation. Since they are all based on search in a template library, by definition, they are unable to predict truly novel sites that have no analogues in their template library (more precisely: in the template database there is no related protein that has a known binding site at a similar location). Template-free methods, on the other hand, rely on intrinsic local properties of protein surface patches or 3D chemical neighourhoods. As such, they can at least potentially predict truly novel binding sites. Whether this limitation will become more or less relevant in the future is an open question. On the one hand, the number of experimentally solved structures grows steadily. Consequently, template databases will improve their coverage of the space of all possible binding sites with time. On the other hand, advances in ab initio protein modeling [54], de novo protein design [55, 56], directed in silico protein evolution [57] and the fact that LBS prediction is being applied to MD trajectories [58] will offer ever more opportunities for novel binding sites to occur.

Another concern related to template based methods is how to meaningfully compare their performance to template-free methods. It is obvious that the query protein structure (for which we want to predict LBS) should be excluded from the template library during evaluation, otherwise the problem is reduced to a simple search. What, then, about very close homologs? To achieve realistic results, authors of eFindSite suggest [45] using sequence identity threshold $t=40 \%$ (35% in earlier work [52]) and excluding templates with higher sequence identity to the query protein when doing benchmarking predictions. This seems reasonable, albeit any particular choice of threshold *t* is inevitably arbitrary. For any method other than eFindSite we can find a particular value *T* for which it will perform roughly the same as eFindSite at $t=T$.

For those reasons we see the two categories of methods as complementary and ideally used in combination where possible; template based methods for their ability to give potentially very high confidence predictions, and template-free methods for the ability to potentially predict truly novel binding sites.

### Prediction speed

Discussion about running times of existing methods has been largely missing in published studies and reviews. See Table 2 for our survey of running times of several web based and stand-alone tools. As it turns out, the differences between times required for prediction by individual methods can be in orders of magnitude.

**Table 2:** Prediction speed

Method	Time^†^
COACH (web server)	15 h (self reported estimate)
eFindSite (web server)	$6.9\pm 0$ h
COACH (stand-alone)	$6.4\pm 2$ h
GalaxySite (web server)	2 h (self reported estimate)
3DLigandSite (web server)	1–3 h (self reported estimate)
ISMBLab-LIG (web server)	$71\pm 2$ min
FTSite (web server)	$39\pm 3$ min
LISE (web server)	$39\pm 0.1$ min
MetaPocket 2.0 (web server)	$2.8\pm 0.4$ min
DeepSite (web server)	$38\pm 0.03$ s
SiteHound (stand-alone)	$12\pm 0.5$ s
P2Rank (stand-alone)	$6.8\pm 0.2$ s (cold start*)
	0.9 s (in larger dataset*)
Fpocket (stand-alone)	$0.2\pm 0.01$ s

^†^Average time required for LBS prediction on a single protein. Displayed is self reported estimate or a result of our test on a small dataset of 5 proteins á $\sim$2500 atoms. Stand-alone tools were tested on a single 3.7 GHz CPU core. For web servers the wall time from submitting a job to receiving the result was measured.

*Difference is due to JVM initialization and model loading cost

But is the speed of prediction even relevant? For use cases involving only a few proteins probably not; after all, it is worth to wait for potentially better predictions. There are use cases, however, for which high computational requirements might be prohibitive. Those include genome-wide structural studies and prediction on trajectories from MD simulations. For illustration, predictions for 40,000 proteins by a stand-alone version of COACH method would take roughly 30 years on a single CPU core (whereas here introduced P2Rank would need only under 12 h).

### Residue-centric versus pocket-centric perspective

Available tools differ also in the way they represent prediction results. Most of the methods produce a ranked list of pockets, which are usually represented as a pocket center and/or as a set of points in the empty space around the protein surface that characterize the shape of the pocket. These could be regularly spaced grid points (most of the methods), alpha sphere centers (Fpocket) or points on a solvent accessible surface (P2Rank). These *pocket-centric* methods are typically evaluated and compared in terms of the identification success rate considering Top-*k* pockets from the ranked list of predicted binding sites (where *k* is usually 1, 3 or 5).

A subset of published methods is focused primarily on predicting ligand binding residues. Many of those methods do not produce a ranked list of binding sites as such, nor do they pinpoint their locations and shapes. Those *residue-centric* methods look at the problem of LBS prediction as to the problem of binary classification of solvent exposed residues to binding and non-binding. This is also the way how they are evaluated and compared, usually in terms of standard binary classification metrics: MCC, AUC or F-measure. This point of view originated with earlier methods for LBS prediction directly from sequence. It is also prevalent as a main evaluation methodology among methods that compete in CASP [59] and CAMEO [60] competitions, where prediction of ligand binding residues on homology models is one of the disciplines.

**Fig. 1:**
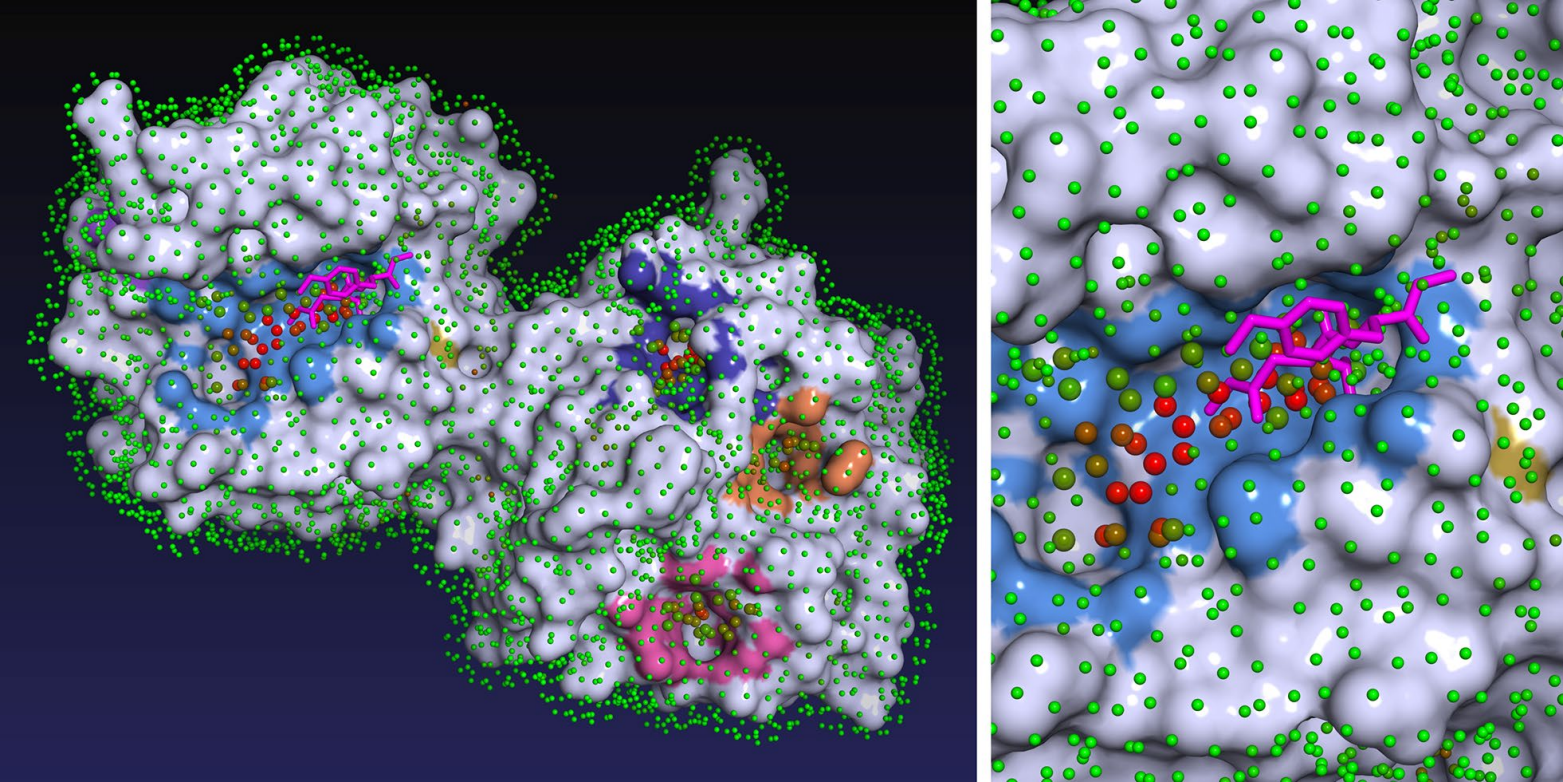
Visualization of ligand binding sites predicted by P2Rank for structure 1FBL. Protein is covered in a layer of points lying on the Solvent Accessible Surface of the protein. Each point represents its local chemical neighborhood and is colored according to its predicted ligandability score (from 0 = green to 1 = red). Points with high ligandablity score are clustered to form predicted binding sites (marked by coloring adjacent protein surface). In this case, the largest predicted pocket (shown in the close-up) is indeed a correctly predicted true binding site that binds a known ligand (magenta). Visualization is based on a PyMOL script produced by P2Rank

This residue-centric view represents not only a different way of looking at the problem, but also a different and in some cases conflicting objective. Methods that are optimized to achieve the best results in binding residue prediction will not necessarily be best at ranked pocket prediction and vice versa. To illustrate where are those objectives misaligned, consider the following case: a method predicts a large binding site centered around a small known ligand, such that predicted pocket defines three times larger protein surface than is the contact surface defined by this known ligand (similar situation can be seen in Fig. 1). How should be this prediction evaluated? From the pocket-centric point of view, it is considered a successful prediction and therefore a net positive. From the residue classification point of view, this adds around twice as much false positives than true positives (2/3 of predicted residues are not contact residues with known ligand) to the confusion matrix, and that will have mostly negative impact toward chosen performance metric. Ligand binding site is a fuzzy concept, even more so is the notion of its exact borders. It is not unreasonable to assume that considered binding site could harbor a larger ligand [61] (perhaps a superstructure of the known small one). It may be objected that this just means that residue-centric view favours more precise predictions. However, by the same token, a residue-centric evaluation methodology will favour spatially precise prediction of one larger binding site over few correct smaller ones.

We believe that pocket-centric point of view better represents a common sense associated with LBS prediction, and as an evaluation methodology awards those methods that fail to predict the least amount of potentially interesting binding sites. In this context, P2Rank is a pocket-centric method.

### Other limitations and advantages of available tools

Available tools have other practical and theoretical limitations. For instance COACH web server limits 3 jobs per user (IP address) and ISMBLab-LIG and eFindSite web servers asks for entering captcha-like code with every prediction request. Some methods are able to predict LBS only on single-chain proteins or they work with single-chain structures internally (this is true for most of the template based methods). This could be a usability inconvenience as preprocessing step of splitting structures by chains is needed first. More importantly, it means that those tools will not be able to predict potential binding sites that emerge around places, where chains connect in multimers and biological assemblies.

It should be acknowledged that some tools offer functionality that goes beyond simple LBS prediction from structure. Some tools are able to perform prediction just from sequence by automatically building a homology model first (GalaxySite, 3DLigandSite, FunFold [23]). Another useful function of some methods is the ability to suggest possible binding ligands (GalaxySite and template based methods). Other tools are able to directly predict druggability of predicted pockets (Fpocket, DogSite) or predict transient pockets in molecular simulation trajectories [62, 63]. That being said, in the present work we assess other tools only by their ability to predict LBS from structure.

## Implementation and usage

P2Rank is a command line program written in Groovy and Java distributed as a binary package that requires no dependencies except Java Runtime Environment. It is lightweight in the sense that (unlike many alternative stand-alone tools) it specifically does not depend on other bioinformatics tools or large structural or sequence databases that would need to be installed on a local machine. It is platform independent (to the extent Java is) and has been tested on Linux and Windows.

Input is a PDB file or a dataset file that contains a list of PDB files. P2Rank is able to automatically produce predictions for any PDB file (single or multi chained) by running a single command (prank predict -f protein.pdb). No preprocessing steps on part of the user are needed. For each input protein, P2Rank produces an output CSV file which contains an ordered list of predicted pockets and their scores. Pockets are characterized by coordinates of their centers, by a list of solvent exposed protein atoms and by a list of amino acid residues that constitute the binding site. PDB file with labeled SAS points (which form a primary internal representation of predicted pockets) can be also produced. The program can optionally generate a PyMOL [64] script that produces 3D visualizations such as the one shown in Fig. 1. In addition to that, P2Rank allows to easily train and evaluate new models on custom datasets and then use them for predictions. This approach can be used to create models that are specialized for specific types of proteins or ligands.

P2Rank has an efficient well optimized implementation: required running time averages to less than 1 s for a protein of $\sim$2500 atoms on a single 3.7 GHz CPU core. On multi-core machines datasets can be processed in parallel with a configurable number of working threads. Memory footprint is around 1GB but grows only slowly with additional working threads. Additionally, P2Rank has a clean internal Java API and apart from being used as a command line tool it can be easily employed as a library for LBS prediction by programs running on JVM.

## Results and discussion

### Results

We have extensively evaluated prediction performance of P2Rank and compared it against several widely used and state-of-the art methods. Those include geometric Fpocket, energetic SiteHound, consensus based MetaPocket 2.0 and deep learning based DeepSite. In the comparison we focused mainly on tools that P2Rank directly competes with: that is template-free stand-alone fully automated tools that are freely available.

It should be noted that our prediction model was trained on CHEN11 dataset and some arbitrary parameters of the algorithm were tweaked with respect to the performance on JOINED dataset (see “Datasets” section). We want to emphasize that only results on CHEN420 and HOLO4K datasets represent an unbiased estimate of P2Rank’s performance.

Results in Table 3 show that P2Rank clearly outperforms other tools in Top-n and Top-(n+2) categories on both datasets. P2Rank also achieves higher success rates that were possible to achieve just by re-scoring predictions of Fpocket by PRANK algorithm (PRANK is part of P2Rank software package and works on similar principles). Still, Fpocket+PRANK performed better than any of the other tools with the exception of P2Rank.

**Table 3:** Comparison of predictive performance on COACH420 and HOLO4K datasets

	COACH420		HOLO4K	
	Top-n	Top-(n+2)	Top-n	Top-(n+2)
Fpocket	56.4	68.9	52.4	63.1
Fpocket+PRANK^a^	63.6	76.5	62.0	71.0
SiteHound^†^	53.0	69.3	50.1	62.1
MetaPocket 2.0^†^	63.4	74.6	57.9	68.6
DeepSite^†^	56.4	63.4	45.6	48.2
P2Rank[protrusion]^b^	64.2	73.0	59.3	67.7
P2Rank	*72.0*	*78.3*	*68.6*	*74.0*

The numbers represent identification success rate [%] measured by DCCcriterion (distance from pocket center to closest ligand atom) with 4 Å threshold considering only pockets ranked at the top of the list (n is the number of ligands in considered structure)

^†^These methods failed to produce predictions for some portion of input proteins. Here we display success rates calculated only based on subsets of proteins, on which they finished successfully. Detailed, pairwise comparison with P2Rank on the exact subsets can be found in the Additional file 1.

^a^Predictions of Fpocket re-scored by PRANK algorithm (which is included in P2Rank software package)

^b^Reduced version of P2Rank that uses only single geometric feature: protrusion

We have also evaluated performance of a reduced version of P2Rank that uses only single geometric feature (descriptor): protrusion. Surprisingly, even this simplified, purely geometric version of P2Rank slightly outperforms other tools in most cases (with the exception of MetaPocket 2.0 in Top-(n+2) category).

Some of the evaluated tools failed to produce predictions on some portion of inputs. Since we wanted to compare the viability of the methods, not just robustness of their implementations, we considered success rates only on subsets of original datasets on which given tools finished successfully and produced predictions. Detailed, pairwise breakdown of the results is included in Additional file 1.

Furthermore, we have compared prediction speed with aforementioned and several additional tools. Results in Table 2 show that P2Rank is faster than other tools with the exception of Fpocket.

Differences in average total number of predicted sites are shown in Table 4. The table also shows that HOLO4K dataset contains larger proteins with more binding sites than COACH420. This is due to the fact that HOLO4K contains mainly multimers and COACH420 only single-chain proteins. Interestingly, Fpocket and P2Rank seem to scale the number of predicted sites with protein size, while MetaPocket 2.0 and DeepSite do not. SiteHound produced significantly more small pockets that other tools.

**Table 4:** Average number of predicted binding sites

	COACH420	HOLO4K
avg. protein atoms	2179	3908
avg. true sites	1.2	2.4
Fpocket	14.6	27
SiteHound	66.2	99.5
MetaPocket 2.0	6.3	6.4
DeepSite	3.2	2.8
P2Rank	6.3	12.6

Displayed is the average total number of binding sites predicted per protein by each method on a given dataset

### Discussion

DeepSite is the only other machine learning based method in our benchmark and we shall discuss how it relates to our method and offer possible explanation for its lower performance. Predictive model of DeepSite is deep convolutional neural network trained on a large dataset of 7622 structures derived from sc-PDB [65] database. DeepSite is based on learning from relatively large instance representations (i.e. model input; $8\times 16^3$ sliding box) and a large dataset, whereas P2Rank is based on smaller representations (1D feature vector) and smaller training dataset. Voxelized representation used by DeepSite, in related works also referred to as *atomic grid* [66, 67], is closer to the raw structural data (atomic coordinates and types) and as such it holds more information. It potentially allows trained model to capture more interactions than our feature based representation. In the light of our results, however, we suspect that even larger training datasets may be needed for such voxelized representations to perform well. Another possible reason for relatively poor performance of DeepSite in our benchmark may be that our respective training sets come form different distributions, more specifically the fact that the relevant ligands (and therefore binding sites) are defined differently. More work is needed to compare respective approaches, ideally using the same training and test datasets and evaluation methodology. This discussion only highlights prevalent and recognized [21, 26, 68] problem of the field: the lack of standardized protocols and benchmarks.

Another general problem in the field is the over-reliance on the ground truth as defined by known protein-ligand complexes from PDB. It is naive to assume that in our datasets all possible binding sites are demarked by bound ligands. That is to say that many locations labeled as negatives (non-binding sites) in the datasets may be binding sites yet to be discovered, or they are already known, but the particular ligand binding is captured in a different PDB entry. Due to protein flexibility and allosteric effects, in some cases it may not even be possible for a protein to bind two ligands at two known binging sites at the same time. We conjecture that between 1/3 and 1/2 of true ligand binding sites are not demarked by ligands in structures directly taken from the PDB. This is particularly problematic for machine learning and knowledge based methods which use such datasets for training their models or constructing their knowledge bases. From their perspective it means that training datasets are extremely noisy.

There is no perfect solution, but the best effort to mitigate this issue we have encountered is expressed in the way CHEN11 dataset was constructed. For all proteins in this dataset, close homologs were found in the PDB, aligned with them and ligands from homologs were superimposed to those structures. Consequently, it is less likely that CHEN11 dataset contains unmarked true binding sites (although some risk that some of those additional binding sites are false is introduced). We believe that this dataset serves as a better source for the ground truth than raw structures taken directly from PDB (therefore we use it as a training set despite its relatively small size). The way this dataset was constructed is akin to the working of template based methods, and we believe that, in a similar way, template based methods can help to construct better training datasets in the future (by adding very high confidence predictions based on close homologs as binding sites).

Furthermore, when such noisy datasets are used for evaluation (of all, not just machine learning based methods), there is a theoretical performance limit that can be achieved even by an optimal predictor (i.e. predictor that achieves Bayes optimal rate). Even optimal predictor would sometimes predict (on top of the ranked list) fundamentally true binding site that is not correctly labeled in the evaluation dataset, with the effect that a 100% success rate would not be achieved on this protein and consequently on the dataset. For this reason we are suspicious when we see reported success rates that are unrealistically high, say close to or above 95% in Top-1/Top-n category (which seem to be above optimal achievable rate on noisy datasets). This can be indicative of a data leakage (in machine learning and knowledge based methods) or overfitting on a given dataset (i.e. dataset was used to optimize parameters during development) or, in case of template based methods, of the fact that the query protein was not removed from the template library during evaluation (as we have seen in some recent papers). We believe that if some method seem to achieve such high success rates, especially on small datasets, it may not be indicative of its true performance and researchers should check for mentioned pitfalls and try to evaluate it on larger datasets. More research is, however, needed to support our conjecture and to provide better estimates.

In the introduction, we have argued that template based methods are not able to predict truly novel sites (with respect to their template library), implying that our method should be better in this regard. A question that can be raised here is, that since our method is based on machine learning from examples, whether that means that it is also only as good as is the training set, and therefore subject to similar limitations as template based methods. The answer is yes, to some extent this is true for any machine learning based method. However, the premise of our method is that the model is not learning to remember particular binding sites, but rather learns what makes local neighbourhoods around the protein surface intrinsically ligandable. Algorithm should then be able to apply this learned generalized knowledge to predict novel sites. But this is exactly what can be illustrated by the performance of our method on a large dataset like HOLO4K.

The unique feature of our method is that we predict ligandability of points on a solvent accessible surface. Other related machine learning approaches were focused on predicting ligandability of residues, solvent exposed atoms or points on a regular grid. In our preliminary experiments, focusing on grid points or atoms led to significantly worse results. We mention it as this insight might be helpful for authors of related methods in the future.

### Future work

One limitation of our tool is that it does not produce exact shapes and volumes of predicted binding sites. For each predicted pocket, P2Rank can produce a set of its SAS points that somewhat define its shape, but they are not regularly spaced in 3D. This is something we would like to address in the future versions of the software, and improve it to produce volumetrically exactly defined, geometrically feasible binding sites. As a consequence, in our evaluation we did not use volumetric overlap identification criteria sometimes employed in other studies [18, 33]. It is possible that other methods produce predictions with more accurate shapes (where those binding sites are found in the first place). However, given the large margin with which P2Rank outperformed other compared methods, it is very unlikely that the conclusions of the benchmark would be different using volumetric criteria.

Currently, P2Rank still does not use all available information that is possible to derive from protein structure. Sequence conservation and energetic calculations (using different probes) could be used to further enrich the feature vector. Our present research is also focused on applying rotation invariant geometric 3D descriptors as well as more powerful machine learning methods to the problem.

## Materials and methods

### P2Rank algorithm

The P2Rank algorithm (which principles we introduced previously in [49]) is based on classification of points evenly spread on protein’s Solvent Accessible Surface (referred to as *SAS points*). These points represent local spherical 3D neighbourhoods that are centered on them. At the same time, they can be seen as potential locations of contact atoms of potential ligands. Initially, SAS points are described by a vector of physico-chemical, geometric and statistical features calculated from its local geometric neighbourhood. Consecutively, a predicted ligandability score is assigned to each SAS point by a machine learning based model. Finally, the points with high predicted ligandability score are clustered to form predicted ligand binding sites (see Fig. 1).

To generate predictions for a given protein using a pre-trained classification model P2Rank follows these instructions:

1. Generate a set of regularly spaced points lying on a protein’s Solvent Accessible Surface (*SAS points*). Positions of the points are calculated by a fast numerical algorithm [69] implemented in CDK library [70].
2. Calculate feature descriptors of SAS points based on their local chemical neighborhood:
  - compute property vectors for protein’s solvent exposed atoms,
  - project distance weighted properties of nearby protein atoms onto SAS points (6Å neighbourhood is considered, $w(d)=1-d/6$),
  - compute additional features describing SAS points’ neighborhoods and assign them directly to SAS points.
3. Predict ligandability score of SAS points by Random Forest classifier.
4. Cluster points with high ligandability score and thus form pocket predictions (single-linkage clustering with 3Å cut-off).
5. Rank predicted pockets by cumulative ligandability score of their points (sum of squared ligandability scores of all points in the cluster).

Initial step of our approach relates our method to the energetic method by Morita et al. [71], where points on a solvent accessible surface were used to discretize space around the protein (in contrast with a typical approach of using points on a regular grid).

Feature vector that represents SAS points and their neighbourhoods contains 35 numerical features, some of which were inspired by other studies [72–76]. For the complete list of features and analysis of their importance, see Additional file 1. The single most important feature turned out to be a geometric feature termed protrusion. It is defined simply as a number of protein atoms within a sphere of 10 Å around a SAS point, and as such can be seen as a proxy for point’s “buriedness”. In the “Results” section we show that even a simplified version of the algorithm, based only on this feature alone, seem to outperform many of the other methods.

P2Rank is distributed with a pre-trained model based on Random Forests algorithm that was trained on a relatively small but diverse CHEN11 dataset (see “Datasets” section). Various arbitrary parameters of the algorithm (cut-offs, thresholds, protrusion radius, etc.) and hyper-parameters of Random Forest were optimized with respect to the performance on JOINED dataset. The final default model has 200 trees, each grown with no depth limit using 6 features.

### Datasets

To train and evaluate P2Rank we were working with following datasets of protein-ligand complexes:

- **CHEN11**—a dataset of 251 proteins harboring 476 ligands introduced in LBS prediction benchmarking study [21]. A non-redundant dataset designed in a way so that every SCOP family [77] has at most one typical representative and to minimize the number of unannotated binding sites (by superimposing ligands from very close homologs). As such it serves as a good source for the ground truth and we employ it as a training set. See [21] for the details on how it was constructed.
- **JOINED**—consists of structures from several smaller datasets used in previous studies (B48/U48, B210, DT198, ASTEX) joined into one larger dataset. We use it as a development set (i.e. validation set).
  - **B48/U48**—Datasets that contain a set of 48 proteins in a bound and unbound state [50].
  - **B210**—a benchmarking dataset of 210 proteins in bound state [50].
  - **DT198**—a dataset of 198 drug-target complexes [27].
  - **ASTEX**—Astex Diverse set [78] is a collection of 85 proteins that was introduced as a benchmarking dataset for molecular docking methods.
- **COACH420**—consists of 420 single chain structures that contain a mix of drug targets and naturally occurring ligands (we have taken COACH test set [42, 43] and removed proteins contained in CHEN11 and JOINED).
- **HOLO4K**—large dataset of protein-ligand complexes based on the list published in [79]. Contains larger multi-chain structures downloaded directly from PDB. Disjunct with CHEN11 and JOINED.

### Evaluation methodology

To evaluate predictive performance of P2Rank and compare it with other methods we have used methodology based on ligand-centric counting and DCC(distance between the center of the pocket and any ligand atom) pocket identification criterion with 4 Å threshold. Binding sites are defined by ligands present in evaluation datasets. Every structure in a dataset can have more than one relevant ligand (see below) and for every relevant ligand, its binding site must be correctly predicted for a method to achieve 100% identification success rate on the given dataset. Every relevant ligand contributes with equal weight toward the final success rate. The output of prediction methods is a ranked list of several putative binding sites, but during evaluation only those ranked at the top are considered. We use Top-*n* and Top-(*n*+2) rank cutoffs where *n* is the number of relevant ligands in the evaluated target protein structure (for proteins with only one ligand this corresponds to the usual Top-1 and Top-3 cutoffs). This evaluation methodology is the same as the one that was used in independent benchmarking study [21]. P2Rank is focused on predicting binding sites for biologically relevant ligands and PDB files in considered datasets often contain ligands (or HET groups) that are not relevant. To determine which ligands are relevant we use a custom filter and alternatively the binding MOAD [80] database. For more details on how we determine which ligands are relevant, see Additional file 1.

## Conclusion

We have presented P2Rank, a novel machine learning based tool for prediction of ligand binding sites from protein structure. We have shown that P2Rank outperforms several alternative tools on two large datasets and that it belongs to the fastest available tools. P2Rank is able to work directly with multi-chain structures and thus find potential binding sites that consist of residues from multiple chains. Among other advantages is the fact that P2Rank works out of the box, as it does not depend on other bioinformatics tools or databases. Unlike many alternative stand-alone tools, P2Rank is able to make fully automated predictions from the command line (no manual preprocessing steps are needed).

P2Rank is, therefore, well suited to be used as a stable component in structural bioinformatics pipelines, where fast and accurate prediction is required. We believe that P2Rank should be particularly beneficial for predicting novel allosteric sites, for which template based methods would generally be less effective. P2Rank is available as an open source command line tool and a Java library.

## Availability and requirements

- Project name: P2Rank
- Project home page: http://siret.ms.mff.cuni.cz/p2rank
- Operating system(s): Platform independent
- Programming language: Groovy, Java
- Other requirements: JRE 8 or higher (Java 1.8)
- Source code: http://github.com/rdk/p2rank
- License: MIT

## Additional file

**Additional file 1.**

## Abbreviations

LBS: ligand binding site(s)
MD: molecular dynamics
PDB: the Protein Data Bank
SAS: solvent accessible surface
MCC: Matthews correlation coefficient
AUC: area under the ROC curve (receiver operating characteristic)
JVM: Java virtual machine

## Footnotes

1 We use the term ‘pocket’ liberally as a convenient one word synonym for ‘ligand binding site’, although not all ligand binding sites are necessarily located in concave pockets.

## References

[1] Konc J, Janežiž D (2014). Binding site comparison for function prediction and pharmaceutical discovery. Curr Opin Struct Biol.

[2] Zheng X, Gan L, Wang E, Wang J (2013). Pocket-based drug design: exploring pocket space. AAPS J.

[3] Pérot S, Sperandio O, Miteva M, Camproux A, Villoutreix B (2010). Druggable pockets and binding site centric chemical space: a paradigm shift in drug discovery. Drug Discov Today.

[4] Tibaut T, Borišek J, Novič M, Turk D (2016). Comparison of in silico tools for binding site prediction applied for structure-based design of autolysin inhibitors. SAR QSAR Environ Res.

[5] Xie L, Xie L, Bourne PE (2011). Structure-based systems biology for analyzing off-target binding. Curr Opin Struct Biol.

[6] Grove Laurie E, Sandor Vajda DK, Fagerberg J, Mowery DC, Nelson RR (2016). Computational methods to support fragment-based drug discovery. Fragment-based drug discovery: lessons and outlook.

[7] Laurie A, Jackson R (2006). Methods for the prediction of protein-ligand binding sites for structure-based drug design and virtual ligand screening. Curr Protein Peptide Sci.

[8] Feinstein WP, Brylinski M (2015). Calculating an optimal box size for ligand docking and virtual screening against experimental and predicted binding pockets. J Cheminform.

[9] Lionta E, Spyrou G, Cournia DKV (2014). Zoe: structure-based virtual screening for drug discovery: principles, applications and recent advances. Curr Top Med Chem.

[10] Schomburg K, Bietz S, Briem H, Henzler A, Urbaczek S, Rarey M (2014). Facing the challenges of structure-based target prediction by inverse virtual screening. J Chem Inf Model.

[11] Degac J, Winter U, Helms V (2015). Graph-based clustering of predicted ligand-binding pockets on protein surfaces. J Chem Inf Model.

[12] Meyers J, Brown N, Blagg J (2016). Mapping the 3D structures of small molecule binding sites. J Cheminform.

[13] Monzon AM, Zea DJ, Fornasari MS, Saldaño TE, Fernandez-Alberti S, Tosatto SCE, Parisi G (2017). Conformational diversity analysis reveals three functional mechanisms in proteins. PLOS Comput Biol.

[14] Shen Q, Cheng F, Song H, Lu W, Zhao J, An X, Liu M, Chen G, Zhao Z, Zhang J (2017). Proteome-scale investigation of protein allosteric regulation perturbed by somatic mutations in 7000 cancer genomes. Am J Hum Genet.

[15] Bhagavat R, Sankar S, Srinivasan N, Chandra N (2018). An augmented pocketome: detection and analysis of small-molecule binding pockets in proteins of known 3D structure. Structure.

[16] Hussein H, Borrel A, Geneix C, Petitjean M, Regad L, Camproux A (2015). PockDrug-Server: a new web server for predicting pocket druggability on holo and apo proteins. Nucleic Acids Res.

[17] Huang W, Lu S, Huang Z, Liu X, Mou L, Luo Y, Zhao Y, Liu Y, Chen Z, Hou T, Zhang J (2013). Allosite: a method for predicting allosteric sites. Bioinformatics.

[18] Le Guilloux V, Schmidtke P, Tuffery P (2009). Fpocket: an open source platform for ligand pocket detection. BMC Bioinform.

[19] Henrich S, Outi S, Huang B, Rippmann F, Cruciani G, Wade R (2010). Computational approaches to identifying and characterizing protein binding sites for ligand design. J Mol Recognit JMR.

[20] Leis S, Schneider S, Zacharias M (2010). In silico prediction of binding sites on proteins. Curr Med Chem.

[21] Chen K, Mizianty M, Gao J, Kurgan L (2011). A critical comparative assessment of predictions of protein-binding sites for biologically relevant organic compounds. Structure (London, England : 1993).

[22] Fauman EB, Rai BK, Huang ES (2011). Structure-based druggability assessment-identifying suitable targets for small molecule therapeutics. Curr Opin Chem Biol.

[23] Roche DB, Brackenridge DA, McGuffin LJ (2015). Proteins and their interacting partners: an introduction to protein-ligand binding site prediction methods. Int J Mol Sci.

[24] Broomhead NK, Soliman ME (2017). Can we rely on computational predictions to correctly identify ligand binding sites on novel protein drug targets? Assessment of binding site prediction methods and a protocol for validation of predicted binding sites. Cell Biochem Biophys.

[25] Simões T, Lopes D, Dias S, Fernandes F, Pereira J, Jorge J, Bajaj C, Gomes A (2017) Geometric detection algorithms for cavities on protein surfaces in molecular graphics: a survey. In: Computer graphics forum 10.1111/cgf.13158 PMC5839519 29520122

[26] Krivak R, Hoksza D (2015). Improving protein-ligand binding site prediction accuracy by classification of inner pocket points using local features. J Cheminform.

[27] Zhang Z, Li Y, Lin B, Schroeder M, Huang B (2011). Identification of cavities on protein surface using multiple computational approaches for drug binding site prediction. Bioinformatics (Oxford, England).

[28] Ghersi D, Sanchez R (2009). EasyMIFS and SiteHound: a toolkit for the identification of ligand-binding sites in protein structures. Bioinformatics (Oxford, England).

[29] Kauffman C, Karypis G (2009). Librus: combined machine learning and homology information for sequence-based ligand-binding residue prediction. Bioinformatics (Oxford, England).

[30] Qiu Z, Wang X (2011). Improved prediction of protein ligand-binding sites using random forests. Protein Peptide Lett.

[31] Chen P, Huang JZ, Gao X (2014). Ligandrfs: random forest ensemble to identify ligand-binding residues from sequence information alone. BMC Bioinform.

[32] Jian JW, Elumalai P, Pitti T, Wu CY, Tsai KC, Chang JY, Peng HP, Yang AS (2016). Predicting ligand binding sites on protein surfaces by 3-Dimensional probability density distributions of interacting atoms. PLoS ONE.

[33] Jiménez J, Doerr S, Martínez-Rosell G, Rose AS, De Fabritiis G (2017). Deepsite: protein-binding site predictor using 3D-convolutional neural networks. Bioinformatics.

[34] Nayal M, Honig B (2006). On the nature of cavities on protein surfaces: application to the identification of drug-binding sites. Proteins.

[35] Halgren TA (2009). Identifying and characterizing binding sites and assessing druggability. J Chem Inf Model.

[36] Capra JA, Laskowski RA, Thornton JM, Singh M, Funkhouser TA (2009). Predicting protein ligand binding sites by combining evolutionary sequence conservation and 3D structure. PLoS Comput Biol.

[37] Wass MN, Kelley LA, Sternberg MJ (2017). 3DLigandSite: predicting ligand-binding sites using similar structures. Nucleic Acids Res.

[38] Yu J, Zhou Y, Tanaka I, Yao M (2010). Roll: a new algorithm for the detection of protein pockets and cavities with a rolling probe sphere. Bioinformatics.

[39] Volkamer A, Griewel A, Grombacher T, Rarey M (2010). Analyzing the topology of active sites: on the prediction of pockets and subpockets. J Chem Inf Model.

[40] Ngan CH, Hall DR, Zerbe B, Grove LE, Kozakov D, Vajda S (2012). FTSite: high accuracy detection of ligand binding sites on unbound protein structures. Bioinformatics.

[41] Xie Z, Hwang M (2012). Ligand-binding site prediction using ligand-interacting and binding site-enriched protein triangles. Bioinformatics.

[42] Roy A, Yang J, Zhang Y (2012). Cofactor: an accurate comparative algorithm for structure-based protein function annotation. Nucleic Acids Res.

[43] Yang J, Roy A, Zhang Y (2013). Protein-ligand binding site recognition using complementary binding-specific substructure comparison and sequence profile alignment. Bioinformatics.

[44] Lee HS, Im W (2013). Ligand binding site detection by local structure alignment and its performance complementarity. J Chem Inf Model.

[45] Brylinski M, Feinstein WP (2013). eFindSite: improved prediction of ligand binding sites in protein models using meta-threading, machine learning and auxiliary ligands. J Comput Aided Mol Des.

[46] Heo L, Shin W, Lee M, Seok C (2014). GalaxySite: ligand-binding-site prediction by using molecular docking. Nucleic Acids Res.

[47] Viet Hung L, Caprari S, Bizai M, Toti D, Polticelli F (2015). Libra: ligand binding site recognition application. Bioinformatics.

[48] Gao J, Zhang Q, Liu M, Zhu L, Wu D, Cao Z, Zhu R (2016). bSiteFinder, an improved protein-binding sites prediction server based on structural alignment: more accurate and less time-consuming. J Cheminform.

[49] Krivák R, Hoksza D (2015) In: Dediu A-H, Hernández-Quiroz F, Martín-Vide C, Rosenblueth AD (eds) P2RANK: knowledge-based ligand binding site prediction using aggregated local features. Springer, Cham, pp 41–52

[50] Huang B, Schroeder M (2006). Ligsitecsc: predicting ligand binding sites using the connolly surface and degree of conservation. BMC Struct Biol.

[51] Laskowski RA, Watson JD, Thornton JM (2005). Profunc: a server for predicting protein function from 3D structure. Nucleic Acids Res.

[52] Brylinski M, Skolnick J (2008). A threading-based method (FINDSITE) for ligand-binding site prediction and functional annotation. Proc Natl Acad Sci USA.

[53] Skolnick J, Brylinski M (2009). FINDSITE: a combined evolution/structure-based approach to protein function prediction. Briefings Bioinform.

[54] Lee J, Freddolino PL, Zhang Y (2017) In: Rigden DJ (ed) Ab initio protein structure prediction. Springer, Dordrecht, pp 3–35

[55] Karanicolas J, Corn J (2011). A de novo protein binding pair by computational design and directed evolution. Mol Cell.

[56] Damborsky J, Brezovsky J (2014). Computational tools for designing and engineering enzymes. Curr Opin Chem Biol.

[57] Wang M, Zhao H (2016) In: Stoddard BL (ed) Combined and iterative use of computational design and directed evolution for protein–ligand binding design. Springer, New York, pp 139–153 10.1007/978-1-4939-3569-7_8 27094289

[58] Di Pietro O, Juárez-Jiménez J, Muñoz-Torrero D, Laughton CA, Luque FJ (2017). Unveiling a novel transient druggable pocket in bace-1 through molecular simulations: conformational analysis and binding mode of multisite inhibitors. PLOS ONE.

[59] Gallo Cassarino T, Bordoli L, Schwede T (2014). Assessment of ligand binding site predictions in CASP10. Proteins Struct Funct Bioinform.

[60] Haas J, Roth S, Arnold K, Kiefer F, Schmidt T, Bordoli L, Schwede T (2013). The protein model portal-a comprehensive resource for protein structure and model information. Database.

[61] Ma B, Shatsky M, Wolfson HJ, Nussinov R (2002). Multiple diverse ligands binding at a single protein site: a matter of pre-existing populations. Protein Sci.

[62] Schmidtke P, Axel B, Luque F, Barril X (2011). MDpocket: open-source cavity detection and characterization on molecular dynamics trajectories. Bioinformatics (Oxford, England).

[63] Stank A, Kokh DB, Horn M, Sizikova E, Neil R, Panecka J, Richter S, Wade RC (2017). Trapp webserver: predicting protein binding site flexibility and detecting transient binding pockets. Nucleic Acids Res.

[64] Schrödinger LLC (2015) The PyMOL molecular graphics system, version 1.8

[65] Desaphy J, Bret G, Rognan D, Kellenberger E (2015). sc-PDB: a 3D-database of ligandable binding sites-10 years on. Nucleic Acids Res.

[66] Ragoza M, Hochuli J, Idrobo E, Sunseri J, Koes DR (2017). Protein-ligand scoring with convolutional neural networks. J Chem Inf Model.

[67] Ragoza M, Turner L, Koes DR (2017) Ligand pose optimization with atomic grid-based convolutional neural networks. ArXiv e-prints

[68] Schmidtke P (2011) Protein-ligand binding sites. Identification, characterization and interrelations. Ph.D. thesis, University of Barcelona

[69] Eisenhaber F, Lijnzaad P, Argos P, Sander C, Scharf M (1995). The double cubic lattice method: Efficient approaches to numerical integration of surface area and volume and to dot surface contouring of molecular assemblies. J Comput Chem.

[70] Steinbeck C, Han Y, Kuhn S, Horlacher O, Luttmann E, Willighagen E (2003). The chemistry development kit (CDK): An open-source Java library for chemo- and bioinformatics. J Chem Inf Comput Sci.

[71] Morita M, Nakamura S, Shimizu K (2008). Highly accurate method for ligand-binding site prediction in unbound state (apo) protein structures. Proteins.

[72] Kyte J, Doolittle RF (1982). A simple method for displaying the hydropathic character of a protein. J Mol Biol.

[73] Desaphy J, Azdimousa K, Kellenberger E, Rognan D (2012). Comparison and druggability prediction of protein-ligand binding sites from pharmacophore-annotated cavity shapes. J Chem Inf Model.

[74] Kapcha LH, Rossky PJ (2014). A simple atomic-level hydrophobicity scale reveals protein interfacial structure. J Mol Biol.

[75] Khazanov NA, Carlson HA (2013). Exploring the composition of protein-ligand binding sites on a large scale. PLoS Comput Biol.

[76] Pintar A, Carugo O, Pongor S (2002). Cx, an algorithm that identifies protruding atoms in proteins. Bioinformatics.

[77] Murzin AG, Brenner SE, Hubbard T, Chothia C (1995). Scop: a structural classification of proteins database for the investigation of sequences and structures. J Mol Biol.

[78] Hartshorn M, Verdonk M, Chessari G, Brewerton S, Mooij W, Mortenson P, Murray C (2007). Diverse, high-quality test set for the validation of protein-ligand docking performance. J Med Chem.

[79] Schmidtke P, Souaille C, Estienne F, Baurin N, Kroemer R (2010). Large-scale comparison of four binding site detection algorithms. J Chem Inf Model.

[80] Hu L, Benson ML, Smith RD, Lerner MG, Carlson HA (2005). Binding moad (mother of all databases). Proteins Struct Funct Bioinform.

[81] Zhu H, Pisabarro MT (2011). MSPocket: an orientation-independent algorithm for the detection of ligand binding pockets. Bioinformatics.

