# Supplementary material for: P2Rank: machine learning based tool for rapid and accurate prediction of ligand binding sites from protein structure
Source: J Cheminform. 2018 Aug 14;10:39. doi: 10.1186/s13321-018-0285-8 (PMC6091426; doi:10.1186/s13321-018-0285-8)
Supplement: Supplementary file 1 — Additional file 1. [file 13321_2018_285_MOESM1_ESM.pdf]

P2Rank: machine learning based tool for rapid and accurate prediction of ligand binding sites from protein structure

Radoslav Krivák and David Hoksza

1 RELEVANT LIGANDS

P2Rank is focused on predicting binding sites for biologically relevant ligands. PDB files in considered datasets often contain more than one such ligand of interest. PDB files also contain a variety of other HET groups like solvents, salt and misplaced groups (which are not in contact with the protein). Instead of declaring only one ligand as relevant for every file in a dataset (as was often done in other ligand binding site prediction studies), we determine relevant ligands by a filter.
Ligands that are considered relevant must comply to these conditions:

- number of ligand atoms is greater or equal than 5
- distance from any atom of the ligand to the closest protein atom is at least 4Å (to remove "floating" ligands)
- distance form the center of the mass of the ligand to the closest protein atom is not greater than 5.5Å (to remove ligands that "stick out")
- name of the PDB group is not on the list of ignored groups:
  `(HOH, DOD, WAT, NAG, MAN, UNK, GLC, ABA, MPD, GOL, SO4, PO4)`

Choosing relevant ligands in exactly this particular way is admittedly arbitrary. In order to make sure our results are robust with respect to the particular way relevant ligands are determined, we have created a versions of JOINED and HOLO4K datasets where relevant ligands are determined in a different way. Binding MOAD [2] release 2013, a database of biologically relevant ligands in PDB, was used to determine relevant ligands in resulting datasets JOINED(Mlig) and HOLO4K(Mlig). PDB files that have no entry in MOAD were removed from the new datasets. It has to be noted that the notion of biologically relevant ligand does not have a widely accepted definition. There are other databases that purportedly collect only biologically relevant ligand interactions from the PDB (e.g. BioLiP [8], PDBbind [7]) that use different criteria for accepting particular ligand as biologically relevant (with MOAD being the strictest of them, not accepting any small ions for example). For the discussion see [8]. We believe that predicting binding sites for ions, peptides and other specific types of binding partners would be better served by specialized methods.

2 ADDITIONAL RESULTS

2.1 Collecting Predictions

P2Rank All reported results correspond to P2Rank v2.0 with default parameters.

Fpocket Stand-alone version of Fpocket v1.0 with default parameters was used (code downloaded from SourceForge repository). Version 2.0RC1 was available at the time but it seemed to be producing consistently worse results.

SiteHound Stand-alone Linux version of SiteHound was downloaded from SiteHound website (version label: January 12, 2010). Command used to generate predictions: `ls *.pdb | xargs -i python ../auto.py -i  -p CMET -k` (executed in directory with pdb files). Default probe and parameters were used.

MetaPocket 2.0 Predictions were obtained from MetaPocket 2.0 web server by web scraping python script in Fall 2017 using default parameters.

DeepSite Predictions were obtained from DeepSite web server by web scraping python script in Fall 2017 using default parameters.

LISE We also made an effort to compare our method with LISE, which is the latest template-free method with a stand-alone version. However, we found that stand-alone version of LISE failed on ∼50% of inputs, mainly due to file

parsing errors. Moreover, on the rest of inputs it exhibited very poor identification success rates (<20%), indicative of some other technical problem. Ultimately, we have decided not to compare results of LISE and P2Rank side by side.

2.2 Detailed Results

Table 1 shows comparison with Fpocket and PRANK, including results on train and validation datasets. Table 2 shows pairwise comparison of P2Rank with SiteHound, MetaPocket 2.0 and DeepSite on exact subsets on which those methods finished successfully and produced predictions.

(Mlig) datasets Tables 1 and 2 also show results on (Mlig) version of the datasets, where relevant ligands were determined in a different way (see Relevant Ligands). Results on (Mlig) datasets tell the same story. In the absolute sense, numbers are higher on HOLO4K(Mlig), which has approx. by 1/3 less relevant binding sites to be predicted than HOLO4K. Nevertheless, P2Rank outperforms other methods with similar margins, especially in Top-n category. Similar margins achieved on those datasets show that our results are robust with respect to the particular way relevant ligands are defined.

Note on DeepSite Presented results of DeepSite on HOLO4K do not represent completely unbiased estimation of its performance. DeepSite is trained on a large dataset which contains some of the proteins that are also included in our test set (733 proteins from HOLO4K), although possibly not on all of the chains.

2.3 Different feature sets

To assess contributions of some features, we have evaluated results of P2Rank with different, reduced, sets of features (Table 3). We would like to note that parameter optimization and final model selection was done with respect to the results on JOINED dataset.

Note on atomic propensity features Atom type propensity features (`apRawValids`,`apRawInvalids`) are based on tables that were calculated from large subset of all protein-ligand complexes from PDB. It is possible that among those complexes were some structures from our test sets. An issue can be raised, that in an absolute sense this may constitute a data leakage; that is to say that there is a possibility that the results reported on those test sets may be biased, as they were achieved with the help of features that were derived also using some structures from those test sets. Practically speaking, contribution of any single protein to numbers in these propensity tables is probably below rounding error. Nevertheless, to avoid possibility of basing our conclusions on biased results, we have evaluated performance of reduced feature set without these propensity features ([full−propensities] in Table 3). Table 3 shows that with respect to the results on COACH420 and HOLO4K, contribution of those features is minimal at best, and on HOLO4K the average success rates without using those features are actually better than results reported in the paper for default P2Rank model. Even if we reported results without using those features, the conclusions of our benchmark and comparison of methods would not change.

Table 1. Comparison with Fpocket and PRANK. Results on CHEN11 (training set) and JOINED (development set) are not representative and are included here only for completeness. In datasets labeled as *Mlig*, relevant ligands (and therefore binding sites that are expected to be predicted) were determined in a different way (see Relevant Ligands).

			Top-n			Top-(n+2)		
Dataset	proteins	ligands	Fpocket	PRANK*	P2Rank	Fpocket	PRANK*	P2Rank
CHEN11	251	476	47.1	**58.2**†	57.9†	57.6	**64.5**†	63.9†
JOINED	537	626	53.8	68.2	**74.4**	72.4	80.0	**80.2**
COACH420	420	511	56.4	63.6	**72.0**	68.9	76.5	**78.3**
HOLO4K	4009	9584	52.4	62.0	**68.6**	63.1	71.0	**74.0**
COACH420(Mlig)	300	378	57.4	64.0	**71.2**	70.4	**76.5**	**76.5**
HOLO4K(Mlig)	3448	6886	56.9	68.3	**73.7**	70.3	79.6	**80.9**

The numbers represent identification success rate [%] measured by D_{CA} criterion (distance from pocket center to closest ligand atom) with 4 Å threshold considering only pockets ranked at the top of the list (n is the number of ligands in considered structure).
*predictions of Fpocket re-scored by PRANK algorithm (which is included in P2Rank software package)
†average results of 10 independent 5-fold cross-validation runs

Table 2. Comparison with SiteHound, MetaPocket 2.0 and DeepSite. Exact pairwise comparison on subsets of the datasets on which compared methods finished successfully. Datasets JOINED/* and HOLO4K/* are subsets of JOINED and HOLO4K on which respective methods finished successfully and produced predictions (SH=SiteHound, MP=MetaPocket2, DS=DeepSite). Similarly for (Mlig) datasets. In datasets labeled as *Mlig*, relevant ligands (and therefore binding sites that are expected to be predicted) were determined in a different way (see Relevant Ligands).

Dataset	proteins	ligands	Top-n		Top-(n+2)	
			SiteHound	P2Rank	SiteHound	P2Rank
COACH420/SH	284	345	53.0	**72.8**	69.3	**77.1**
HOLO4K/SH	2878	6826	50.1	**68.8**	62.1	**74.3**
COACH420(Mlig)/SH	203	257	51.0	**70.4**	67.7	**75.1**
HOLO4K(Mlig)/SH	2470	4843	53.1	**74.0**	67.8	**81.3**
			MetaPocket 2.0	P2Rank	MetaPocket 2.0	P2Rank
COACH420/MP	417	508	63.4	**72.2**	74.6	**78.1**
HOLO4K/MP	2575	5021	57.9	**72.4**	68.6	**77.7**
COACH420(Mlig)	300	378	62.2	**71.2**	73.3	**76.5**
HOLO4K(Mlig)/MP	2202	3706	62.3	**78.3**	75.2	**84.6**
			DeepSite	P2Rank	DeepSite	P2Rank
COACH420	420	511	56.4	**72.0**	63.4	**78.3**
HOLO4K/DS	3991	9557	45.6	**68.6**	48.2	**74.0**
COACH420(Mlig)	300	378	54.5	**71.2**	61.6	**76.5**
HOLO4K(Mlig)/DS	3430	6861	50.8	**73.7**	54.4	**80.8**

The numbers represent identification success rate [%] measured by D_{CA} criterion (distance from pocket center to closest ligand atom) with 4 Å threshold considering only pockets ranked at the top of the list (n is the number of ligands in considered structure).

Table 3. Predictive performance of different feature sets. The numbers represent identification success rate [%] measured by D_{CA} criterion (distance from pocket center to closest ligand atom) with 4 Å threshold considering only pockets ranked at the top of the list (n is the number of ligands in considered structure). In rows representing feature sets each number is an average results of 10 train/eval runs.

	JOINED		COACH420		HOLO4K	
	Top-n	Top-(n+2)	Top-n	Top-(n+2)	Top-n	Top-(n+2)
[protrusion][a]	62.8	73.4	64.2	73.0	59.3	67.7
[full−protrusion][b]	64.3	75.9	60.5	71.8	68.2	75.9
[full−propensities][c]	73.9	80.5	71.6	77.9	69.1	74.7
[full][d]	74.0	80.2	71.4	78.1	70.1	75.4
P2Rank (default model)[e]	74.4	80.2	72.0	78.3	68.6	74.0

[a]reduced set of features that includes only one feature: protrusion
[b]reduced set of features that does not include protrusion
[c]reduced set of features that does not include atomic propensity features (see "ap*" features)
[d]full set of features
[e]Default pre-trained model of P2Rank (with full set of features). Note that numbers are slightly different from [full] since this row represents the results of a particular pre-selected model (the default model P2Rank is distributed with), while [full] row contains averages of 10 runs. Model selection was done based on performance on JOINED.

3 FEATURES

Features that are used to describe accessible surface points are listed in Table 4.

Table 4. - Complete list of features that are used to describe solvent accessible surface (SAS) points. *Type: a...values are assigned to protein solvent exposed atoms and then projected onto SAS points p...values are assigned directly to SAS points **source: values are determined by Amino Acid Type table / Atom Type table / given in PDB file / calculated on the spot

Feature name	T*	source**	description
`hydrophobic`	a	AA tab.	binary attribute, 1 for hydrophobic residues
`hydrophilic`	a	AA tab.	binary attribute, 1 for hydrophilic residues
`hydrophatyIndex`	a	AA tab.	side-chain hydropathy index with values in range $\langle -4.5, 4.5 \rangle$ [5]
`aliphatic`	a	AA tab.	binary attribute, 1 for aliphatic residues
`aromatic`	a	AA tab.	binary attribute, 1 for aromatic residues
`sulfur`	a	AA tab.	binary attribute, 1 for residues containing sulfur
`hydroxyl`	a	AA tab.	binary attribute, 1 for hydroxyl group containing residues
`basic`	a	AA tab.	binary attribute, 1 for basic residues
`acidic`	a	AA tab.	binary attribute, 1 for acidic residues
`amide`	a	AA tab.	binary attribute, 1 for amide group containing residues
`posCharge`	a	AA tab.	binary attribute, 1 for positively charged residues
`negCharge`	a	AA tab.	binary attribute, 1 for negatively charged residues
`hBondDonor`	a	AA tab.	binary attribute, 1 for H-bond donor containing residues
`hBondAcceptor`	a	AA tab.	binary attribute, 1 for H-bond acceptor containing residues
`hBondDonorAcceptor`	a	AA tab.	binary attribute, 1 for residues that have H-bond donor AND acceptor
`polar`	a	AA tab.	binary attribute, 1 for polar residues
`ionizable`	a	AA tab.	binary attribute, 1 for ionizable residues
`vsAromatic`	a	AT tab.	VolSite atomic level features [1]
`vsCation`	a	AT tab.	
`vsAnion`	a	AT tab.	
`vsHydrophobic`	a	AT tab.	
`vsAcceptor`	a	AT tab.	
`vsDonor`	a	AT tab.	
`atomicHydrophobicity`	a	AT tab.	Atom type hydrophobicity scale [3]
`apRawValids`	a	AT tab.	Ligand binding propensity for biologically valid ligands [4]
`apRawInvalids`	a	AT tab.	Ligand binding propensity for biologically invalid ligands [4]
`bfactor`	a	given	B-factor number of the atom from pdb file
`atoms`	p	calc.	absolute number of protein exposed atoms in the neighbourhood (within 6 Å radius of the point)
`atomDensity`	p	calc.	number of protein exposed atoms weighted by distance
`atomC`	p	calc.	number of carbon atoms in the neighbourhood
`atomO`	p	calc.	number of oxygen atoms in the neighbourhood
`atomN`	p	calc.	number of nitrogen atoms in the neighbourhood
`hDonorAtoms`	p	calc.	number of H-bond donor atoms in the neighbourhood
`hAcceptorAtoms`	p	calc.	number of H-bond acceptor atoms in the neighbourhood
`protrusion`	p	calc.	Protein surface protrusion inspired by [6] calculated simply as number of all protein atoms (not just exposed) within 10 Å radius of the point

3.1 Feature Importances

Table 5 contains calculated feature importances.

Table 5. Feature Importances.

feature	importance
`protrusion`	0.084528
`bfactor`	0.013888
`apRawInvalids`	0.011785
`vsAromatic`	0.010165
`apRawValids`	0.009403
`atomO`	0.009275
`hydrophobic`	0.008630
`hydrophilic`	0.007643
`vsAcceptor`	0.006244
`vsHydrophobic`	0.005273
`atoms`	0.005188
`aromatic`	0.004433
`atomN`	0.004236
`hydrophatyIndex`	0.004232
`atomC`	0.003687
`vsDonor`	0.003451
`aliphatic`	0.003350
`atomicHydrophobicity`	0.002663
`hBondDonorAcceptor`	0.002650
`hDonorAtoms`	0.002626
`atomDensity`	0.002549
`polar`	0.002402
`ionizable`	0.002142
`hAcceptorAtoms`	0.001904
`hBondAcceptor`	0.001705
`sulfur`	0.001621
`negCharge`	0.001538
`acidic`	0.001504
`basic`	0.001467
`hydroxyl`	0.001328
`vsAnion`	0.001072
`hBondDonor`	0.001059
`posCharge`	0.001021
`vsCation`	0.000832
`amide`	0.000831

Feature importances calculated by Random Forest algorithm on CHEN11 dataset. Avg. of 10 runs.

REFERENCES

[1] J. Desaphy, K. Azdimousa, E. Kellenberger, and D. Rognan. Comparison and druggability prediction of protein-ligand binding sites from pharmacophore-annotated cavity shapes. *Journal of chemical information and modeling*, 52(8):2287–2299, 2012.

[2] L. Hu, M. L. Benson, R. D. Smith, M. G. Lerner, and H. A. Carlson. Binding moad (mother of all databases). *Proteins: Structure, Function, and Bioinformatics*, 60(3):333–340, 2005.

[3] L. H. Kapcha and P. J. Rossky. A simple atomic-level hydrophobicity scale reveals protein interfacial structure. *Journal of Molecular Biology*, 426(2):484 – 498, 2014.

[4] N. A. Khazanov and H. A. Carlson. Exploring the composition of protein-ligand binding sites on a large scale. *PLoS computational biology*, 9(11):e1003321, Nov 2013.

[5] J. Kyte and R. F. Doolittle. A simple method for displaying the hydropathic character of a protein. *Journal of Molecular Biology*, 157(1):105 – 132, 1982.

[6] A. Pintar, O. Carugo, and S. Pongor. Cx, an algorithm that identifies protruding atoms in proteins. *Bioinformatics*, 18(7):980–984, 2002.

[7] R. Wang, X. Fang, Y. Lu, and S. Wang.

[8] J. Yang, A. Roy, and Y. Zhang. Biolip: a semi-manually curated database for biologically relevant ligandprotein interactions. *Nucleic Acids Research*, 41(D1):D1096–D1103, 2013.
